# EHMTI-0188. Contraceptive-induced amenorrhoea leads to reduced migraine frequency in women with menstrual migraine

**DOI:** 10.1186/1129-2377-15-S1-G21

**Published:** 2014-09-18

**Authors:** EA MacGregor, KG Vetvik, C Lundqvist, MB Russell

**Affiliations:** 1 Blizard Institute of Cell and Molecular Science, Barts and the London School of Medicine and Dentistry, London, UK; 2 Institute of Clinical Medicine, University of Oslo, Oslo, Norway

## Introduction

Menstrual migraine without aura (MM) is defined as attacks of migraine without aura (MO) occurring on day 1±2 of the menstrual cycle in at least 2/3 menstruations. Population-based studies shows that MM affects approximately 20% of female migraineurs in their 30s. Many women in this age group need contraception.

## Aim

The aim of the present study was to investigate the influence of hormonal contraception on attacks of migraine without aura (MO) in women with a history of MM.

## Methods

A total of 237 women from the general population with self-reported migraine in at least half of their menstruations were interviewed about headache and course of headache during use of hormonal contraception by a neurologist. Among these, 141 women had a history of MM according to the International Classification of Headache Disorders II.

## Results

Among 141 women with a history of MM, 49 women were currently using hormonal contraception. Of these, 23 reported amenorrhoea and the remaining reported withdrawal bleeds/menstruation. Significantly more women with amenorrhoea reported no MO-attacks during the preceding month compared to women without amenorrhoea (OR 16.1; 95% confidence interval (CI) 1.8-140.4; P = 0.003). A reduction of MO-frequency was more often reported in women with than without amenorrhoea (OR 3.5; 95% CI 1.1-11.4; P = 0.04). Pain intensity and attack duration did not differ between the groups.

## Conclusion

Amenorrhoea leads to a reduction of MO-frequency in women with MM using hormonal contraceptives. Future prospective studies on MM should focus on contraceptive methods that achieve amenorrhoea.

No conflict of interest.

